# Early-Life Factors and Early-Onset Endometrial Cancer Risk in the UK Biobank

**DOI:** 10.1001/jamanetworkopen.2024.40181

**Published:** 2024-10-15

**Authors:** Noah C. Peeri, Kelli O’Connell, Elizabeth D. Kantor, V. Wendy Setiawan, Xingyi Guo, Loren Lipworth, Mengmeng Du

**Affiliations:** 1 Department of Epidemiology and Biostatistics, Memorial Sloan Kettering Cancer Center, New York, New York; 2 Department of Population and Public Health Sciences, University of Southern California, Los Angeles; 3 Norris Comprehensive Cancer Center, University of Southern California, Los Angeles; 4 Division of Epidemiology, Department of Medicine, Vanderbilt University Medical Center, Nashville, Tennessee

## Abstract

This case-control study investigates UK Biobank data for 8 early-life factors and early-onset endometrial cancer risk among UK residents.

## Introduction

Carcinogenesis can span decades, and early-life factors may influence cancers in younger adults.^1^ Endometrial cancer (EC) is rising most rapidly in women younger than 50 years (early onset) in the UK and US.^2,3^ The well-established link with age at menarche suggests that the etiologically relevant window for EC may start early in life.^4,5^ However, little is known about early-life exposures and EC development. To generate hypotheses, we explored 8 early-life factors and early-onset EC risk in the UK Biobank.

## Methods

The UK Biobank is a prospective cohort of 502 366 UK residents (eMethods in Supplement 1) approved by the North West Multicenter Research Ethics Committee. Participants provided written informed consent. In this case-control study, we defined early-onset cases as females at least 18 years of age with EC diagnosed before the age of 50 years^2,6^ either before baseline (prevalent) or during follow-up (incident). We do not anticipate that this biased results as early-life factors occurred before diagnosis. Controls were females with an intact uterus and no history of invasive cancer at baseline, except nonmelanoma skin cancer. To include all eligible participants, we did not match controls to cases. We excluded individuals missing all early-life exposures, leaving 162 prevalent cases, 24 incident cases, and 203 345 controls. We followed the STROBE reporting guideline.

We evaluated 8 early-life factors: comparative body size at age 10 years, comparative height at age 10 years, maternal smoking at birth, age at menarche, breastfed as a child, birth by cesarean delivery, number of older biological siblings, and recurrent childhood/adolescent antibiotic use. We used prespecified categories as self-reported in the UK Biobank questionnaire. For statistical efficiency, we categorized self-reported race as White or other race because approximately 95% of our population reported as White. Weight was characterized by UK Biobank terms plumper and thinner.

We used minimally adjusted (age, race, household income) and fully adjusted (additionally adjusted for family history of cancer, early-life factors) multivariable logistic regression to estimate odds ratios (ORs) and 95% CIs for early-life factors and early-onset EC risk. We performed 2-sided tests using α = .05. Statistical analysis was performed from March 2023 to July 2024 using R statistical software version 4.3.1.

## Results

We included 186 patients with early-onset EC and 203 345 control individuals aged 39 to 71 years (Table 1). Patients with EC more often had published endometrial risk factors, such as obesity, diabetes, nulliparity, and family history of cancer.^4,5^ Plumper body size (vs thinner) at age 10 years was associated with increased early-onset EC risk after multivariable adjustment (OR, 1.56 [95% CI, 1.02-2.41]) (Table 2). Average height (vs shorter) at age 10 years was positively associated with risk (OR, 1.56 [95% CI, 1.04-2.43]), but taller height was not associated. Associations were attenuated after adjusting for adult body mass index (BMI) (plumper vs thinner BMI: OR, 1.27 [95% CI, 0.81-1.98]) and height (average vs shorter height: OR, 1.50 [95% CI, 0.96-2.41]). Age at menarche of at least 12 years (vs ≤11 years) was associated with lower risk (age of menarche at 12-13 years: OR, 0.50 [95% CI, 0.35-0.70]; ≥14 years: OR, 0.52 [95% CI, 0.36-0.74]). Remaining early-life factors were not associated. Restricting analyses to incident early-onset ECs did not alter interpretations.

**Table 1.** Participant Characteristics

Characteristic	No. (%)	
	Participants with EC (n = 186)	Controls (n = 203 345)
Age at baseline, y		
35 to <50	62 (33.3)	56 753 (27.9)
50 to <60	50 (26.9)	71 560 (35.2)
60 to <70	72 (38.7)	74 318 (36.5)
≥70	2 (1.1)	714 (0.4)
Race^a^		
White	176 (94.6)	192 078 (94.5)
Other	10 (5.4)	11 267 (5.5)
Household income, $		
<18 000	45 (24.2)	38 000 (18.7)
18 000-30 999	38 (20.4)	43 266 (21.3)
31 000-51 999	31 (16.7)	44 878 (22.1)
52 000-100 000	26 (14.0)	34 699 (17.1)
>100 000	11 (5.9)	9235 (4.5)
Do not know/prefer not to answer/missing	35 (18.8)	33 267 (16.4)
BMI		
Normal/underweight (<25)	60 (32.6)	85 189 (42.1)
Overweight (25-<30)	48 (26.1)	72 882 (36.0)
Obese (30-<35)	39 (21.2)	29 537 (14.6)
Severely obese (≥35)	37 (20.1)	14 865 (7.3)
Smoking		
Never	117 (63.6)	122 142 (60.2)
Previous	51 (27.7)	62 647 (30.9)
Current	16 (8.7)	17 946 (8.9)
Oral contraceptive use		
No	52 (28.0)	35 767 (17.6)
Yes	134 (72.0)	167 140 (82.4)
Parity		
0	74 (39.8)	40 584 (20.0)
1-2	81 (43.5)	115 980 (57.1)
≥3	31 (16.7)	46 633 (22.9)
Diabetes		
No	170 (91.4)	196 344 (96.7)
Yes	16 (8.6)	6638 (3.3)
Menopausal status		
No	22 (12.0)	61 039 (31.7)
Yes	66 (35.9)	131 682 (68.3)
Not sure, had a hysterectomy	96 (52.2)	0
Postmenopausal hormone use		
No	114 (62.0)	140 447 (69.3)
Yes	70 (38.0)	62 326 (30.7)
Family history of cancer		
No	92 (49.5)	116 549 (57.3)
Yes	83 (44.6)	69 790 (34.3)
Missing	11 (5.9)	17 006 (8.4)
Early life factors^b^		
Comparative body size at age 10 y		
Thinner	43 (23.1)	63 924 (31.4)
About average	99 (53.2)	103 530 (50.9)
Plumper	44 (23.7)	35 891 (17.7)
Missing	0	0
Comparative height at age 10 y		
Shorter	27 (14.5)	43 352 (21.3)
About average	112 (60.2)	108 725 (53.5)
Taller	47 (25.3)	51 268 (25.2)
Missing	0	0
Maternal smoking		
No	122 (65.6)	128 327 (63.1)
Yes	41 (22.0)	49 815 (24.5)
Missing	23 (12.4)	25 203 (12.4)
Age at menarche, y		
≤11	63 (33.9)	37 593 (18.5)
12-13	65 (34.9)	87 072 (42.8)
≥14	54 (29.0)	73 023 (35.9)
Missing	4 (2.2)	5657 (2.8)
No. of older biological siblings		
0	29 (15.6)	28 796 (14.2)
1	21 (11.3)	25 668 (12.6)
≥2	13 (7.0)	21 129 (10.4)
Missing	123 (66.1)	127 752 (62.8)
Breastfed		
No	55 (29.6)	51 115 (25.1)
Yes	103 (55.4)	116 449 (57.3)
Missing	28 (15.1)	35 781 (17.6)
Recurrent childhood/adolescent antibiotic use		
No	54 (29.0)	58 569 (28.8)
Yes	10 (5.4)	12 483 (6.1)
Missing	122 (65.6)	132 293 (65.1)
Birth via cesarean delivery		
No	59 (31.7)	73 968 (36.4)
Yes	4 (2.2)	2066 (1.0)
Missing	123 (66.1)	127 311 (62.6)

Abbreviations: BMI, body mass index (calculated as weight in kilograms divided by height in meters squared); EC, endometrial cancer.

^a^ For statistical efficiency we analyzed self-reported race as White and other (Asian or Asian British, Black or Black British, Chinese, Mixed, other) because nearly 95% of participants identified as White in our population. The UK Biobank assessed self-reported race as Asian or Asian British, Black or Black British, Chinese, Mixed, White, and other. UK Biobank does not provide further granularity for participants in the other race group.

^b^ Most self-reported early-life factors were asked of all participants at baseline. Number of older biological siblings was introduced as a baseline variable midway through the recruitment period (April 2009). Long-term and/or recurrent antibiotic use as a child and/or teenager and birth by cesarean delivery were only asked of a subset of participants during online follow-up in 2017.

**Table 2.** Association Between Early-Life Factors and Early-Onset EC Risk

Characteristic	Early-onset EC			
	No. cases (%)	No. controls (%)	OR (95% CI)	
			Minimally adjusted^a^	Fully adjusted^b^
Comparative body size at age 10 y				
Thinner	43 (23.1)	63 924 (31.4)	1 [Reference]	1 [Reference]
About average	99 (53.2)	103 530 (50.9)	1.43 (1.01-2.07)	1.28 (0.90-1.87)
Plumper	44 (23.7)	35 891 (17.7)	1.82 (1.19-2.77)	1.56 (1.02-2.41)
*P* value for trend	NA	NA	.005	.04
Comparative height at age 10 y				
Shorter	27 (14.5)	43 352 (21.3)	1 [Reference]	1 [Reference]
About average	112 (60.2)	108 725 (53.5)	1.65 (1.10-2.56)	1.56 (1.04-2.43)
Taller	47 (25.3)	51 268 (25.2)	1.49 (0.93-2.42)	1.30 (0.82-2.13)
*P* value for trend	NA	NA	.20	.40
Maternal smoking at birth				
No	122 (74.8)	128 327 (72.0)	1 [Reference]	1 [Reference]
Yes	41 (25.2)	49 815 (28.0)	0.85 (0.59-1.21)	0.82 (0.56-1.16)
Age at menarche				
≤11	63 (34.6)	37 593 (19.0)	1 [Reference]	1 [Reference]
12-13	65 (35.7)	87 072 (44.1)	0.49 (0.35-0.69)	0.50 (0.35-0.70)
≥14	54 (29.7)	73 023 (36.9)	0.48 (0.33-0.69)	0.52 (0.36-0.74)
*P* value for trend	NA	NA	<.001	.007
No. of older siblings^c^				
0	29 (46.0)	28 796 (38.1)	1 [Reference]	1 [Reference]
1	21 (33.3)	25 668 (34.0)	0.80 (0.45-1.41)	0.81 (0.45-1.41)
≥2	13 (20.7)	21 129 (27.9)	0.59 (0.30-1.12)	0.57 (0.29-1.09)
*P* value for trend	NA	NA	.09	.08
Breastfed as child				
No	55 (34.8)	51 115 (30.5)	1 [Reference]	1 [Reference]
Yes	103 (65.2)	116 449 (69.5)	0.84 (0.60-1.18)	0.85 (0.61-1.19)
Recurrent childhood/adolescent antibiotic use^c^				
No	54 (84.4)	58 569 (82.4)	1 [Reference]	1 [Reference]
Yes	10 (15.6)	12 483 (17.6)	0.85 (0.41-1.61)	0.83 (0.40-1.56)
Birth by cesarean delivery^c^				
No	59 (93.7)	73 968 (97.3)	1 [Reference]	1 [Reference]
Yes	4 (6.3)	2066 (2.7)	2.40 (0.73-5.86)	2.31 (0.70-5.65)

Abbreviation: EC, endometrial cancer; NA, not applicable; OR, odds ratio.

^a^ Minimally adjusted analysis adjusted for age, race, and household income.

^b^ Fully adjusted models were additionally adjusted for family history of any cancer, and other early-life factors.

^c^ Analysis was restricted to the subset of participants with this data available (n = 71 116 antibiotic use; n = 76 097 birth by cesarean delivery; n = 75 656 number of older siblings). In fully adjusted models adjusting for these factors, we included a missing data indicator. We tested for trend across ordinal variables.

## Discussion

In this case-control study of early-life factors, larger body size and average height in childhood were associated with approximately 1.6-fold higher risk of early-onset EC. Although statistically significant, CIs were wide, and associations require confirmation. Adult BMI and height may have mediated these associations. Consistent with prior data, older age at menarche was associated with lower risk.^4,5^ Remaining factors may not play a prominent role. Certain early-life factors may help mark EC risk decades later.

Confounding by traditional risk factors was less likely because exposures occurred early in life. The UK Biobank is not EC-specific and early-life exposures are not well-known cancer risk factors; this likely reduced potential for recall bias and exposure misclassification more likely attenuated findings. We did not have information on familial syndromes; adjustment for family history of cancer did not alter results. UK Biobank did not collect childhood weight; however, childhood comparative body size may be more accurately recalled than weight. Sample size was limited to detect modest associations.

Certain early-life factors, including body size, height, and age at menarche were strongly associated with early-onset EC risk in our population. These data underscore the need for research to understand the role of early-life factors to help combat the rapid increase in EC in younger women.

## Notes

Supplement 1. **eMethods.** Supplemental Methods

Supplement 2.
Data Sharing Statement
