# Supplementary material for: Early-Life Factors and Early-Onset Endometrial Cancer Risk in the UK Biobank
Source: JAMA Netw Open. 2024 Oct 15;7(10):e2440181. doi: 10.1001/jamanetworkopen.2024.40181 (PMC11581484; doi:10.1001/jamanetworkopen.2024.40181)
Supplement: Supplement 1. — eMethods. Supplemental Methods [file jamanetwopen-e2440181-s001.pdf]

Supplemental Online Content

Peeri NC, O'Connell K, Kantor ED, et al. Early-life factors and early-onset endometrial cancer risk in the UK Biobank. *JAMA Netw Open.* 2024;7(10):e2440181. doi:10.1001/jamanetworkopen.2024.40181

eMethods. Supplementary Methods

This supplemental material has been provided by the authors to give readers additional information about their work.

© 2024 Peeri NC et al. *JAMA Network Open.*

eMethods. Supplementary Methods

Study Population

The UK Biobank is a large prospective cohort of over 500,000 UK residents.[1] Participants aged 40-69 years were recruited between 2006-2010 across 22 centers in Scotland, England and Wales. UK Biobank participant assessments included 5 parts: 1) Written consent; 2) touch screen questionnaires; 3) face-to-face interview with a study nurse; 4) biometric measurements; and 5) biospecimen collection. For this analysis, most early-life factors were assessed at baseline via questionnaire. Number of older biological siblings was introduced as a baseline variable midway through the recruitment period (April 2009). Long-term/recurrent antibiotic use as a child/teenager and birth by C-section were only asked of a subset of participants during online follow-up in 2017. Lifestyle and medical history were assessed at baseline via questionnaire and interviews. On joining the UK Biobank, participants' health status is followed up over time (generally annually) via linkage to their health-related records and registries. This includes hospital inpatient admissions records, primary care records, and cancer and death registry data.

In this analysis, we excluded males (n=218,949), females with a hysterectomy (n=47,279), and participants missing ≥5% early life exposure data (n=5,992). We used a missing indicator for covariates missing ≥5%.

Because long-term/recurrent antibiotic use as a child/teenager and birth by C-section were only asked of a subset of participants in online follow-up in 2017, we conducted analyses with these

© 2024 Peeri NC et al. *JAMA Network Open.*

variables in the subset of participants for whom data were available (n=71,116 and n=76,097, respectively). Additionally, because number of older siblings was only asked in the latter half of the recruitment period (April 2009), we included individuals with available data on this variable in subsequent analysis (n=75,656).

Statistical Analysis

We used multivariable logistic regression to estimate odds ratios (OR) and 95% confidence intervals (CI) for the association between early-life factors and risk of early-onset endometrial cancer. We used minimally (age, race, income) and fully (further including family history of cancer and all early-life factors) adjusted models. We tested for trend across ordinal variables.

We largely assessed covariates at the baseline survey between 2006 and 2010. Three variables were only collected in a subset of the population: Number of older biological siblings was introduced as a variable midway through the recruitment period (April 2009); long-term/recurrent antibiotic use as a child/teenager and birth by C-section were only asked of a subset of participants during online follow-up in 2017. We have largely used a complete case approach, in which analyses include only participants with complete data (ie, those missing covariate data were excluded). In fully-adjusted models we have alternatively used a missing indicator for variables missing >5% data (e.g., income, family history of cancer, number of older biological siblings, antibiotic use as a child/teenager, birth by C-section, maternal smoking, breastfed as child). This approach avoids the issue of model nonconvergence due to small cell counts (resulting from small numbers missing for most covariates), but also avoids excluding a large number of participants because of missing data for a particular covariate.

© 2024 Peeri NC et al. *JAMA Network Open.*

Although power was very limited, we conducted sensitivity analysis using minimally-adjusted multivariable Cox regression to examine hazard ratios for the association between early-life factors and risk of incident endometrial cancer (we were not powered to conduct fully adjusted multivariable Cox regression).

1. UK Biobank. UK Biobank. https://www.ukbiobank.ac.uk/

© 2024 Peeri NC et al. *JAMA Network Open.*
