# Supplementary material for: Early-Life Factors and Early-Onset Endometrial Cancer Risk in the UK Biobank
Source: JAMA Netw Open. 2024 Oct 15;7(10):e2440181. doi: 10.1001/jamanetworkopen.2024.40181 (PMC11581484; doi:10.1001/jamanetworkopen.2024.40181)
Supplement: Supplement 2. — Data Sharing Statement [file jamanetwopen-e2440181-s002.pdf]

Data Sharing Statement

Peeri. Early-Life Factors and Early-Onset Endometrial Cancer Risk in the UK Biobank. *JAMA Netw Open*. Published October 15, 2024. doi:10.1001/jamanetworkopen.2024.40181

Data
Data available: No
